# Treatment Response to Oncolytic Virus in Patient-Derived Breast Cancer and Hypopharyngeal Cancer Organoids: Evaluation via a Microfluidics Organ-on-a-Chip System

**DOI:** 10.3390/bioengineering12020146

**Published:** 2025-02-04

**Authors:** Yu Sun, Jiaqi Liu, Li Zhu, Fang Huang, Yanbo Dong, Shuang Liu, Siyi Chen, Wei Ji, Jingjing Lu, Liangfa Liu, Shanhu Li

**Affiliations:** 1 Department of Otolaryngology and Head and Neck Surgery, Beijing Friendship Hospital, Capital Medical University, Beijing 100050, China; sunyuoreo@163.com (Y.S.); dennis3d@ccmu.edu.cn (Y.D.);; 2 Department of Cell Engineering, Beijing Institute of Biotechnology, Beijing 100071, China; cc12310754572@163.com (J.L.);; 3 Department of General Surgery, The First Medical Center, Chinese PLA General Hospital, 28 Fuxing Road, Haidian District, Beijing 100853, China; 4 Medical School of Chinese PLA, Beijing 100853, China

**Keywords:** oncolytic virus, patient-derived organoids, breast cancer, head and neck tumors, microfluidics, organ-on-a-chip

## Abstract

In this study, we present an oncolytic virus (OV) evaluation system established using microfluidic organ-on-a-chip (OOC) systems and patient-derived organoids (PDOs), which was used in the development of a novel oncolytic virus, AD4-GHPE. An OV offers advantages such as good targeting ability and minimal side effects, and it has achieved significant breakthroughs when combined with immunotherapy in recent clinical trials. The development of OVs has become an emerging research focus. PDOs can preserve the heterogeneity of in situ tumor tissues, whereas microfluidic OOC systems can automate and standardize various experimental procedures. These systems have been applied in cutting-edge drug screening and cell therapy experiments; however, their use in functionally complex oncolytic viruses remains to be explored. In this study, we constructed a novel recombinant oncolytic adenovirus, AD4-GHPE, and evaluated OOC systems and PDOs through various functional validations in hypopharyngeal and breast cancer organoids. The results confirmed that AD4-GHPE exhibits three antitumor mechanisms, namely, tumor-specific cytotoxicity, a reduction in programmed death ligand 1 (PD-L1) expression in tumor cells to increase CD8^+^ T-cell activity, and granulocyte–macrophage colony-stimulating factor (GM-CSF) secretion. The evaluation system combining OOC systems and PDOs was efficient and reliable, providing personalized OV treatment recommendations for patients and offering industrialized and standardized research ideas for the development of OVs.

## 1. Introduction

Cancer remains the leading cause of death globally; cancer treatment is an immense healthcare burden and, thus, an urgent biomedical issue [1,2,3]. Immunotherapy and biologic therapies, including oncolytic virus (OV) therapy, have become new strategies for cancer treatment [4,5,6]. OV therapy targets tumor cells, replicating inside them and causing lysis, and genetically engineered OVs can complement the antitumor mechanisms of immunotherapy. Combining both methods has become a new research focus in cancer treatment [7,8,9,10,11,12,13]. OV modification can be achieved by adding nucleic acid fragments or short hairpin RNA (shRNA) fragments that regulate protein expression in the tumor microenvironment, thereby killing tumors or enhancing antitumor immune responses [14,15,16]. For example, OVs with specific shRNAs can specifically inhibit programmed death ligand 1 (PD-L1) expression in tumor cells, enhancing T-cell antitumor activity. Furthermore, genes can be added to increase the expression of proteins such as GM-CSF, IL-17, and CXCL9 in the tumor microenvironment, increasing immune cell activity [17,18,19,20]. OVs with complex structures and diverse functions are becoming increasingly common.

Clinical studies on OVs have revealed substantial variation in their therapeutic efficacy among patients, possibly due to tumor heterogeneity. Patient-derived organoids (PDOs) [16,21], which can be generated from various types of human tumors, preserve the heterogeneity and spatial structure of the primary tumor. PDOs are commonly used in drug screening for cancer patients and provide personalized drug recommendations [22,23,24,25]. Organ-on-a-chip (OOC) technology, a novel cell culture method that uses miniature devices, can display physiological behaviors and drug responses more conveniently and in a standardized manner [26,27,28,29]. The use of microfluidic organ chips can improve the standardization and industrialization of PDO evaluation, and these chips have been applied in drug screening and cell therapy [30,31,32,33,34,35]. However, they have not yet been applied in the development of functionally complex OVs.

In a previous study, Rhee et al. reported the use of microprecision 3D printing technology to construct chip models [36]. Compared with other OOCs used for 3D culture, the microchannel network can simulate a uniform nutrient supply and waste removal by capillaries from cells within the chip. The design of the chip scaffold reduces learning costs and facilitates batch processing, which is particularly suitable for PDO culture and OV development.

In this study, we integrated microfluidic OOC technology with breast and hypopharyngeal cancer PDO models to develop OVs. We present a novel OV, AD4-GHPE, constructed by replacing the E3 region with the telomerase reverse transcriptase promoter (TERTp). This virus targets tumors and carries PD-L1-shRNA and GM-CSF sequences. This modification enables AD4-GHPE to exhibit tumor-specific cytotoxicity, reduce PD-L1 expression in tumor cells to enhance T-cell immune responses and secrete GM-CSF for antitumor activity. We cultured PDOs from breast and hypopharyngeal cancer surgical specimens and validated them via tumor marker and hematoxylin and eosin (H&E) staining, confirming that the PDOs exhibited characteristics of the primary tumor. We subsequently demonstrated that AD4-GHPE has tumor-specific cytotoxicity and enhances CD8^+^ T-cell immune responses by reducing PD-L1 expression in breast and hypopharyngeal cancer PDOs.

To reduce manmade systematic errors and improve the efficiency of AD4-GHPE development, we established a microfluidic OOC evaluation system for OV development. On the basis of the high-precision printing technology of Boston Micro Fabrication (BMF), we made local improvements to previous chips, allowing the microchannel network in the chip to more uniformly supply nutrients to PDOs and simulate the diffusion of OVs through the tumor microvasculature. We repeated AD4-GHPE specific cytotoxicity experiments on PDO and CD8^+^ T-cell coculture experiments using this system. Reducing the number of operational steps and time involved produced consistent results, confirming the system’s feasibility. Furthermore, we used this system to demonstrate that AD4-GHPE can significantly increase the expression of GM-CSF in the culture medium. In conclusion, we demonstrate that AD4-GHPE excels in three antitumor mechanisms and that the microfluidic OOC system has significant potential in the development of complex-functioning OVs.

## 2. Materials and Methods

### 2.1. Human Specimens

The specimens used for the experimental PDOs were obtained from tumor tissues and adjacent normal tissues from surgery patients at Beijing Friendship Hospital (Batch number: 2018-P2-198-01), Capital Medical University, and the Chinese PLA General Hospital (Batch number: S2024-675-02). All experiments involving human samples received approval from the necessary ethical committee. Written informed consent was obtained from all patients before sample collection. The samples were confirmed as tumor tissues through pathologist evaluation.

### 2.2. Adenovirus Construction, Preparation, and Identification

Recombinant OV construction strategy: The plasmid pBR322-Ad4-WT(SBI, Palo Alto, CA, USA), containing the complete genome of adenovirus type 4, was modified by multiple enzyme digestions and ligation. The cytomegalovirus (CMV) promoter-controlled GM-CSF gene was added after the inverted terminal repeat (ITR) packaging signal. The original E1A promoter was deleted and replaced with the human telomerase promoter (TERTp). Part of the E3 region sequence was deleted, and a SpeI single restriction enzyme site was added. Finally, the shPD-L1 RNA sequence controlled by the H1 promoter was inserted into the SpeI enzyme site. Enzyme digestion and sequencing were performed for each step for confirmation to obtain the correct recombinant plasmid, pAD4-GHPE. The pAD4-GHPE plasmid was digested with AsiSI enzyme (3811, 34221; NEB, Ipswich, USA), and the 34221 bp DNA fragment was recovered via ethanol precipitation. This construct was then transfected into AD293 cells to rescue the virus. When the transfected cells became swollen and rounded, showing a cytopathic effect (CPE), the cells were collected and subjected to three freeze–thaw cycles. After centrifugation, the supernatant containing the viral stock was transferred to a new EP tube. The viral stock was then transfected into A549 cells for amplification. After CPE occurred in the cells, they were collected and subjected to three freeze–thaw cycles again. The cell lines AD293, 293T, and LO2 were purchased from American Type Culture Collection (ATCC, Manassas, USA). 2BS cells (human embryonic lung fibroblasts, telomerase negative) were purchased from the National Collection of Authenticated Cell Cultures (NCAC, Shanghai, China). Primary head and neck cancer cells (66T, 68T) were isolated and cultured in our laboratory, and cervical cancer cells (C33A), melanoma cells A375, and lung cancer cells A549 were purchased from the National Experimental Cell Resource Sharing Platform (NICR, Beijing, China).

### 2.3. Organoid Culture

The tissues were processed accordingly as previously described [37]: the surgical specimens were cut into small pieces and digested with 1 mg/mL collagenase IV (Sigma–Aldrich, St. Louis, MO, USA) in adDMEM/F12 medium (Gibco, Waltham, MA, USA) and gently shaken at 37 °C. After digestion, the sample was filtered through a 70-μm cell strainer into a fresh 15-mL centrifuge tube. The sample was centrifuged at 800 rpm for 5 min to collect the cells. Culture medium and Matrigel (Corning, NY, USA) were mixed at a ratio of 3:4 and added to the cells. Then, 1 × 10^5^ isolated cells were added to 70 µL of the mixture and added to the center of a 24-well plate. Afterward, 500 µL of the corresponding culture medium was added, and the cells were cultured.

### 2.4. Microfluidic OOC

The chip and scaffold were sourced from BMF Nano Material Technology Co. (BMF Nano Material Technology Co., Shenzhen, China). As previously described [36], the chip was designed using SolidWorks (Dassault Systèmes, Vélizy-Villacoublay, France) and fabricated via a BMF microArch S230 projection micro stereolithography printer (BMF, Maynard, MA, USA). The square-shaped perfusable microchannel features an inner edge size of 500 µm and a wall thickness of 50 µm. In this experiment, the BMF increased the number of 7 µm micropores on the chip to three rows. Prior to the initiation of the experiment, the chip was meticulously cleaned using 70% isopropyl alcohol and subsequently subjected to ultraviolet light irradiation for a duration of 30 min to ensure proper curing. The sterilized chip was fitted with an o-ring on the inlet and then clipped into the bottom half of the scaffold, which was designed to fit into a 6-well plate. Following this, the top half of the chip scaffold was placed atop the bottom half. Tubing and needles were connected to the inlet and outlet ports on the lid, establishing a reliable connection to their corresponding syringes. Two independently operable microfluidic pumps were employed to precisely regulate the injection and aspiration processes of the syringes. These pumps control the flow of medium through the chip by simultaneously injecting medium through the inlet and aspirating waste through the outlet. The inlet flow rate was set to 200 µL/h and the outlet flow rate was set to 250 µL/h (slightly faster than the inlet) to prevent leakage. The low flow rate was 40 µL/h, and the outlet flow rate was 50 µL/h.

### 2.5. Organoid Immunofluorescence

As previously described [38], the organoid culture medium in the 24-well plate was removed, and the plate was centrifuged at 4 °C and 400× *g* to remove the Matrigel. The organoids were treated with 4% paraformaldehyde, Triton X-100, BSA, and Tween 20 (Sigma–Aldrich) and incubated with primary antibodies for 6 h. Antibodies against HER-2, ER, PR, α-Tubulin, and PD-L1 were obtained from Proteintech (Proteintech, St. Pearl, IL, USA). Antibodies against P63 and KRT-5 were obtained from Santa Cruz Biotechnology (Santa Cruz Biotechnology, Santa Cruz, TX, USA). After washing, fluorescence-conjugated secondary antibodies (Invitrogen, Thermo Fisher Scientific, Waltham, MA, USA) and DAPI (Invitrogen, Thermo Fisher Scientific) were added. The organoids were resuspended and transferred to a 3.5 cm laser confocal cell culture dish. Images were acquired via the imageXpresse Micro Confocal objective (Molecular Devices, San Jose, CA, USA). Image analysis was conducted via IN Carta software (V2.5) (Molecular Devices).

### 2.6. Hematoxylin–Eosin Staining and Identification of PDOs

Hematoxylin–eosin (H&E) staining and identification of PDOs were conducted as previously described [39]. Briefly, 1 mL of phosphate-buffered saline (PBS) was used to collect PDOs in 24-well plates, and Matrigel drops were transferred to a 15 mL centrifuge tube containing 10 mL of cold PBS. The organoids were then allowed to settle via gravity and washed. Subsequently, 4 mL of 4% paraformaldehyde (PFA) was added to fix the organoids overnight. The tissue was embedded in paraffin and sectioned into 5 μm thick slices for H&E staining.

### 2.7. Western Blotting

Proteins were isolated from cancer cell suspensions subjected to different treatments, and protein concentrations were measured via a DC protein assay kit (Bio-Rad, Hercules, CA, USA). Equal concentrations of protein were resolved via SDS–PAGE, and blotting was conducted via the Bio-Rad V3 Western Workflow system (Hercules, CA, USA). The PVDF membranes were blocked with TBST (containing 5% BSA) and incubated overnight at 4 °C with the primary antibody PD-L1 (Proteintech). Protein expression was quantified via a densitometer and normalized to that of α-tubulin (housekeeping protein) for comparison.

### 2.8. Enhanced Green Fluorescent Protein Detection

After PDOs were digested into single cells, they were infected with E3-eGFP. In a 96-well plate, a total of 25,000 PDOs were added to 20,000 plaque-forming units (pfu)/well of virus. Enhanced green fluorescent protein (eGFP) fluorescence was observed after 72 h using an Olympus IX51 microscope (wavelength 480 nm) (Olympus, Tokyo, Japan). eGFP signals in individual organoids were quantified via ImageJ software (V1.8.0.112, RRID: SCR_003070) (National Institutes of Health, Hercules, Bethesda, USA), and the percentage of the stained area was expressed as the mean ± SEM.

### 2.9. In Vitro Tumor Killing Assay

After PDO dissociation, 5000 cells per well were mixed with 40% culture medium and 60% Matrigel and added to a 96-well plate in 10 µL units. After the Matrigel solidified, 100 µL of fresh growth medium was added, and the cells were cultured for 48 h until the organoids regained their spherical shape. After 48 h, the medium was replaced with fresh medium containing a specified dose of virus. After 96 h, CellTiter-Glo (Promega, Madison, WI, USA) was added to assess performance. In the microfluidics system, 100 µL of the same Matrigel mixture (containing 50,000 cells from the PDOs) was added to each chip. The microfluidics system was connected, and fresh medium was perfused at a low flow rate while keeping the bidirectional microfluidic pump open. After 48 h, the recovery microfluidic pump was closed, and the medium containing a specified dose of oncolytic virus was perfused for 5 h. After 96 h, the recovery pump was opened, and the supernatant was collected for GM-CSF ELISA detection. CellTiter-Glo (Promega) was added to assay cell activity according to the manufacturer’s instructions.

### 2.10. CD8^+^ T-Cell Culture and Sorting

Peripheral blood mononuclear cells (PBMCs) were separated via Ficoll (Solarbo, Beijing, China) gradient centrifugation. CD8^+^ T cells were stimulated and activated via ImmunoCult (Stemcell, Cambridge, UK) and cultured in X-VIVO 15 medium (Lonza, Basel, Switzerland) for 7 days. CD8^+^ T cells were sorted via CD8^+^ T-positive selection magnetic beads (Miltenyi, San Diego, CA, USA). After sorting, 2 × 10^5^ cells were immediately used for flow cytometry analysis.

### 2.11. Flow Cytometry

The following anti-human antibodies were purchased from BioLegend (San Diego, CA, USA): CD3, CD4, CD8, and CD56. Flow cytometry analysis was performed via a BD Canto II (BD Biosciences, San Jose, CA, USA), and data were acquired and analyzed via BD FACSDiva software (BD Biosciences).

### 2.12. CD8^+^ T Cell Co-Culture Experiment

After the PDOs were dissociated, 5000 cells per well were mixed with 60% organoid culture medium and 40% Matrigel and added to a 96-well plate in 10 µL units. After the Matrigel solidified, 100 µL of fresh growth medium was added, and the organoids were cultured for 48 h until they recovered their spherical morphology. After 48 h, the supernatant was aspirated, and 10 µL of the Matrigel mixture containing 5000 sorted CD8^+^ T cells was added. After fixation, fresh medium containing a specified dose of virus was added, and the cells were cultured for 96 h. The supernatant was collected for IFN-γ detection via ELISA. In the microfluidics system, 100 µL of the same Matrigel mixture was combined with 50,000 dissociated PDO cells and added to the chip. After the Matrigel solidified, the microfluidics system was connected to infuse fresh medium at a low flow rate, and the dual-direction microfluidic pumps were kept open. After 48 h, the recovery pump was turned off, and 50,000 CD8^+^ T cells were added. The medium containing the specified oncolytic virus concentration was then infused at a standard flow rate for 5 h. After 96 h, the recovery pump was turned on to rapidly collect the supernatant for IFN-γ detection via ELISA

### 2.13. ELISA Detection

The supernatant was collected from the samples and centrifuged at 800× *g* for 15 min at 4 °C. Cytokine quantification was performed via IFN-γ and GM-CSF ELISA kits (DAKEWE, Shenzhen, China) per the manufacturer’s instructions. In brief, samples were collected and centrifuged at 800× *g* for 15 min at 4 °C, and detection was conducted per the manufacturer’s instructions. ELISA data were obtained via a Varioskan Flash (Thermo Fisher Scientific).

### 2.14. Statistical Analyses

The data are expressed as the means ± standard deviations (SDs). Statistical analyses were performed via GraphPad Prism (V9.5.0) (GraphPad Software, San Diego, CA, USA). Comparisons between two groups were performed via two-tailed paired *t* tests (and nonparametric tests) and 95% confidence intervals (CIs). In the figures, ns, *, **, ***, and **** represent *p* ≥ 0.05, *p* < 0.05, *p* < 0.01, *p* < 0.001, and *p* < 0.0001, respectively.

## 3. Results

### 3.1. AD4-GHPE Oncolytic Virus Construction

**Figure 1:**
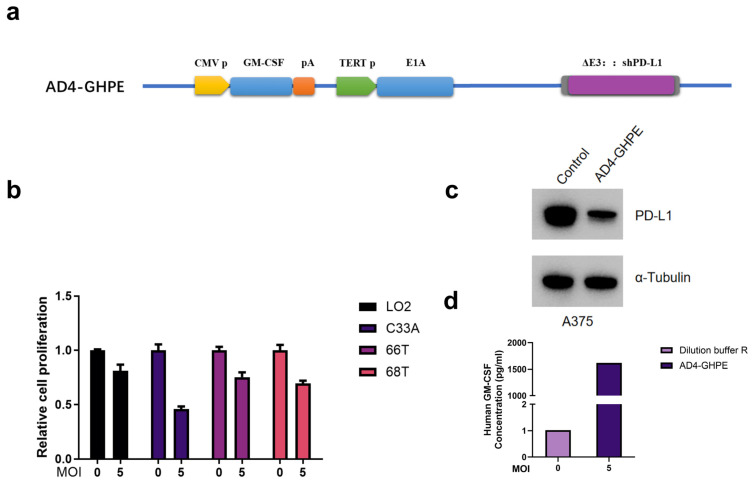
Construction and Preliminary Functional Evaluations of the Oncolytic Adenovirus. (**a**) Schematic diagram of the AD4-GHPE oncolytic adenovirus structure. (**b**) The AD4-GHPE virus was used to infect primary head and neck cancer cells (66T, 68T), cervical cancer cells (C33A), and normal human cells (LO2) at a multiplicity of infection (MOI) of 5. After 72 h, cell viability was measured. (**c**) A549 cells were inoculated in cell culture plates with AD4-GHPE at an MOI of 5; culture medium was collected 48 h after infection, and GM-CSF expression was measured by ELISA. (**d**) A549 cells were infected in cell culture plates with AD4-GHPE at an MOI of 0 or 5; culture medium was collected 48 h after infection, and GM-CSF expression was measured by ELISA.

We constructed a novel OV, AD4-GHPE, via adenovirus type 4. In the recombinant adenovirus we developed, the E3 region of the adenovirus genome was deleted, and a promoter (telomerase reverse transcriptase promoter, TERTp) that is selectively expressed in tumor cells was used to control the expression of the E1A gene, which is essential for adenovirus replication. This modification allows the OV to specifically replicate in tumor cells. Moreover, an antitumor immune-enhancing cytokine, GM-CSF, was expressed in the upstream region of the adenovirus E1 area. In the E3-deleted region, an immune checkpoint inhibitor regulatory shRNA was inserted to prevent tumor immune suppression (Figure 1a). To preliminarily investigate the specific cytotoxicity of AD4-GHPE and its ability to reduce the surface expression of tumor PD-L1 and secretion of GM-CSF, we conducted experiments in 2D cell lines. The results showed that the recombinant AD4-GHPE virus exhibited strong cytotoxicity against primary head and neck and cervical cancer cells, with no significant cytotoxic effect on normal LO2 cells (Figure 1b). After infection with the AD4-GHPE recombinant virus, PD-L1 protein expression was significantly reduced in human melanoma A375 cells (Figure 1c). The AD4-GHPE virus was able to express GM-CSF in large quantities and secrete it into the culture medium. The concentration of GM-CSF in the culture medium was 1617.8 pg/mL, as determined by ELISA (Figure 1d).

### 3.2. Cultivation and Identification of Breast Cancer and Hypopharyngeal Squamous Cell Carcinoma PDOs

**Figure 2:**
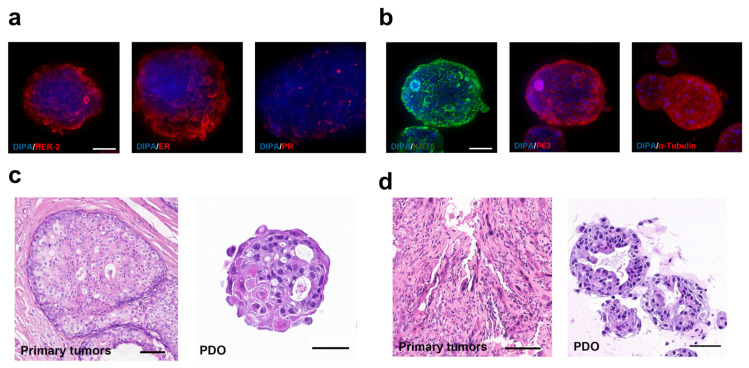
Tumor Organoid Identification. (**a**) In the breast cancer PDOs, both HER-2 and ER demonstrated strong immunoreactivity, while PR was not expressed, consistent with the phenotypic characteristics of primary breast cancer. Scale bar: 100 μm. (**b**) In hypopharyngeal cancer organoids, tumor basal cells labeled with KRT5 and P63 were observed, with α-tubulin staining showing no specificity, matching the distribution of cells in the primary tumor. Scale bar: 40 μm. (**c**,**d**) H&E staining of breast cancer and hypopharyngeal cancer tumor organoid sections compared with those of primary tumors. Scale bars: 100 μm, 50 μm, 100 μm and 100 μm.

We cultivated three breast tumor organoids and three head and neck squamous cell carcinoma organoids, along with corresponding normal tissue organoids, from surgical specimens from different patients. The breast cancer-derived tumor PDOs were named BC-1, BC-2, and BC-3, and the hypopharyngeal squamous cell carcinoma-derived tumor PDOs were named PhC-1, PhC-2, and PhC-3. During surgery, normal tissues adjacent to the tumors were also resected. To study the specificity of the AD4-GHPE oncolytic virus, we cultured organoids from normal tissue specimens of four patients and named them Br-1, Br-2, Ph-1, and Ph-2, which correspond to the same tumor PDO numbers. To confirm the origin and tumor characteristics of the PDOs, we used immunofluorescence staining to detect the expression of specific tumor markers [40,41] in both types of tumor PDOs. H&E staining was used to compare the histological characteristics of PDOs with those of primary tumor sites. The results revealed that in breast cancer PDOs, HER-2 and estrogen receptor (ER) exhibited strong immunoreactivity, whereas progesterone receptor (PR) expression was lower, consistent with the phenotype of primary breast cancer (Figure 2a). In hypopharyngeal carcinoma organoids, tumor basal cells were labeled with P63 (tumor protein 63) and KRT5 (keratin 5), and α-tubulin unspecifically labeled the entire structure, matching the phenotype of the primary hypopharyngeal carcinoma (Figure 2b). H&E staining revealed that the organoids resembled the primary tumors in terms of morphology, such as cellular dysplasia and irregular nuclei (Figure 2c,d), further confirming the tumor characteristics of the organoids.

### 3.3. Specific Replication and Cytotoxicity

**Figure 3:**
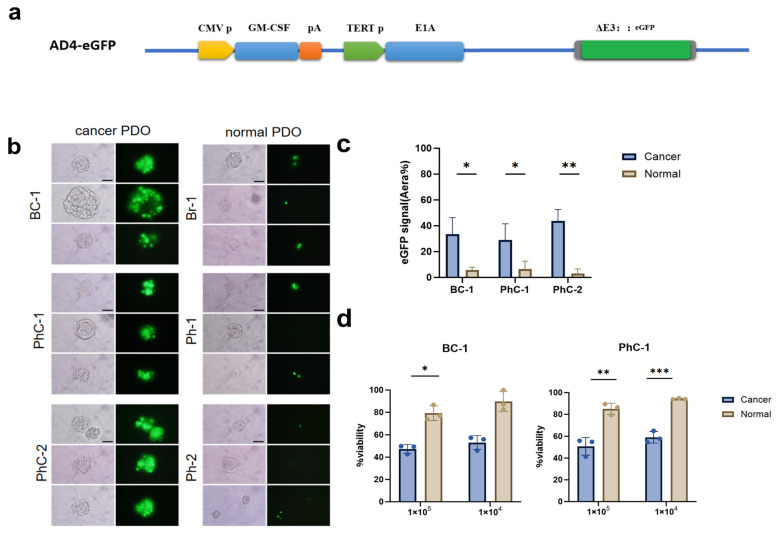
AD4-GHPE Oncolytic Virus Exhibits Specific Infection of Tumor Organoids (**a**) E3-eGFP oncolytic adenovirus profile. (**b**) The TrypLE enzyme was used to dissociate PDOs into small pieces, and then 1 × 10^5^ pfu of E3-eGFP was added. Representative images of eGFP expression 72 h after addition. Scale bar: 100 μm. (**c**) Quantification of eGFP fluorescence, expressed as the percentage of the fluorescence area relative to the total area of the bright-field image of individual organoids. The data are shown as the means ± SEMs (*n* ≥ 3); * *p* < 0.05, ** *p* < 0.01, *t* test. (**d**) AD4-GHPE cytotoxicity in different organoids in vitro. AD4-GHPE was used to infect BC-2 and PhC-1 tumor organoids and their corresponding normal tissue organoids at 1 × 105 pfu/well or 1 × 104 pfu/well in 100 μL of medium. After 96 h, cell viability was assessed via the CellTiter-Glo^®^ 3D assay. The data are presented as the means ± SEMs, n = 3; * *p* < 0.05, ** *p* < 0.01, *** *p* < 0.001, *t* test.

We constructed an AD4-eGFP virus to study the specific replication of the oncolytic virus in tumor PDOs. To maintain the same tumor specificity as AD4-GHPE, we replaced the E3 region shRNA of AD4-GHPE with eGFP (Figure 3a). We conducted AD4-eGFP-specific replication experiments on three tumor PDOs (BC-1, PhC-1, and PhC-2) and their corresponding normal tissue organoids. The replication of the AD4-eGFP oncolytic virus differed between tumor PDOs and normal tissue PDOs (Figure 3b). The fluorescence area in BC-1, PhC-1, and PhC-2 was larger than that in their corresponding normal organoids (Figure 3c), indicating specific infection by AD4-eGFP. Notably, after 72 h of infection with AD4-eGFP, the tumor-derived PDOs presented significant morphological changes, such as edge bulging and lysis, whereas the normal tissue PDOs maintained relatively intact spherical structures. To further confirm the specific cytotoxicity of AD4-GHPE in tumor PDOs, we conducted experiments on two groups of PDOs (BC-1 and PhC-1) and their corresponding normal tissue organoids. AD4-GHPE exhibited cytotoxicity in both BC-1 and PhC-1 cells (Figure 3d). In BC-1 and PhC-1 cells, the viability of the 1 × 10^5^ pfu group decreased by 5.79% and 2.38%, respectively, compared with those of the 1 × 10^4^ pfu group, indicating that the cytotoxicity of AD4-GHPE is potentially dose-dependent. The cytotoxicity of AD4-GHPE to tumor PDOs was significantly higher than that observed in normal tissue organoids. In the 1 × 10^4^ pfu experimental group with a relatively low virus concentration, the survival rates of tumor PDOs in the BC-1 and PhC-1 groups decreased by 33.35% and 41.82%, respectively, compared with those of normal tissue PDOs. This result further confirmed the specificity of this oncolytic virus in killing tumor cells.

### 3.4. PD-L1 Expression Downregulation and Enhanced Immune Response of CD8^+^ T Cells

**Figure 4:**
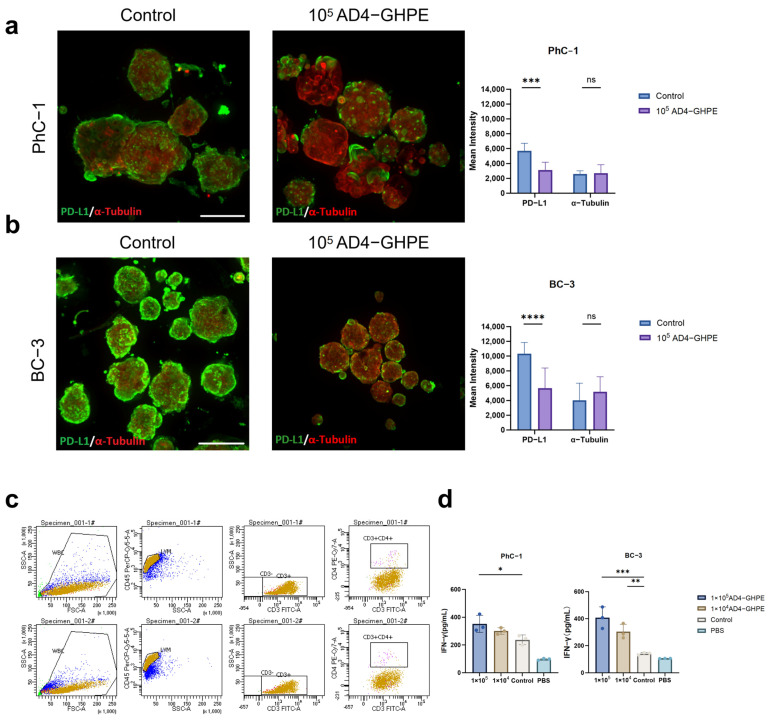
AD4-GHPE Oncolytic Virus shRNA Enhances CD8^+^ T Cell Immune Response by Reducing PD-L1 Expression. (**a**,**b**) Left: control group and experimental group, expression of α-tubulin (red) and PD-L1 (green). In the experimental group, tumor organoids were cultured with 5 × 10^5^ pfu of AD4-GHPE for 72 h. Scale bar: 220 μm. (**a**,**b**) Right: Fluorescence quantification of each organoid image; FITC: PD-L1; TRITC: α-tubulin. Image analysis was conducted via IN Carta software(V2.5). The data are shown as the means ± SEMs, *n* ≥ 7, ns ≥ 0.05, *** *p* < 0.05, **** *p* < 0.0001, *t* test. (**c**) PBMCs were sorted using CD8^+^ T-cell magnetic beads, followed by flow cytometry analysis, with a CD8^+^ T-cell proportion greater than 94%. (**d**) After the digestion of PDOs and CD8^+^ T cells into single cells, 5000 organoids and 5000 CD8^+^ T cells per well were cocultured in 100 μL of medium containing 1 × 10^5^ pfu/well, 1 × 10^4^ pfu/well, or no AD4-GHPE oncolytic virus for 96 h. IFN-γ expression was measured in the cells and supernatants via ELISA. The data are shown as the means ± SEMs, *n* = 3, * *p* < 0.05, ** *p* < 0.05, *** *p* < 0.001, *t* test.

The AD4-GHPE oncolytic virus carries PD-L1 shRNA. Enzymes such as dicer in the cytoplasm process shRNA into small interfering RNA (siRNA). The siRNA interferes with protein synthesis by silencing messenger RNA (mRNA) and ultimately knocks down PD-L1 expression. We previously demonstrated this characteristic in cell line experiments through Western blotting (Figure 1c). In the PDO experiments, we further confirmed this characteristic via immunofluorescence in BC-3 and PhC-1 cells (Figure 4a). Quantitative fluorescence analysis revealed a significant reduction in the green fluorescence intensity of PD-L1, whereas the red fluorescence intensity of α-tubulin was not significantly different (Figure 4b). The results of the immunofluorescence quantitative analysis showed the same trend as the Western blotting data shown in Figure 1c. To further explore the enhancement of the T-cell immune response by AD4-GHPE, we conducted a coculture experiment of CD8^+^ T cells and tumor PDOs with the AD4-GHPE oncolytic virus. After isolating PBMCs from peripheral blood and selecting CD8^+^ T cells via magnetic beads, we performed flow cytometry (Figure 4c) and revealed that highly purified CD8^+^ T cells were obtained. IFN-γ is a hallmark cytokine produced by CD8^+^ T cells and is involved in the antitumor immune response [42,43,44,45]. The level of expression reflects the activation status of T cells. PDO cells, CD8^+^ T cells, and specific concentrations of the AD4-GHPE virus were added to each well of a 96-well plate. After 96 h, ELISA was used to measure IFN-γ expression in the supernatant and cells (Figure 4d). The results showed that in BC-3 and PhC-1 cells, the IFN-γ expression levels in the experimental groups with 1 × 10^5^ AD4-GHPE were 312.50 pg/mL and 407.59 pg/mL, respectively, which were 2.17 times and 2.89 times that of the control group. The IFN-γ expression levels in the experimental groups with 1 × 10^4^ AD4-GHPE were also higher than those in the control group. This finding indicates that AD4-GHPE enhanced the activity of CD8^+^ T cells.

### 3.5. Microfluidic OOCs

**Figure 5:**
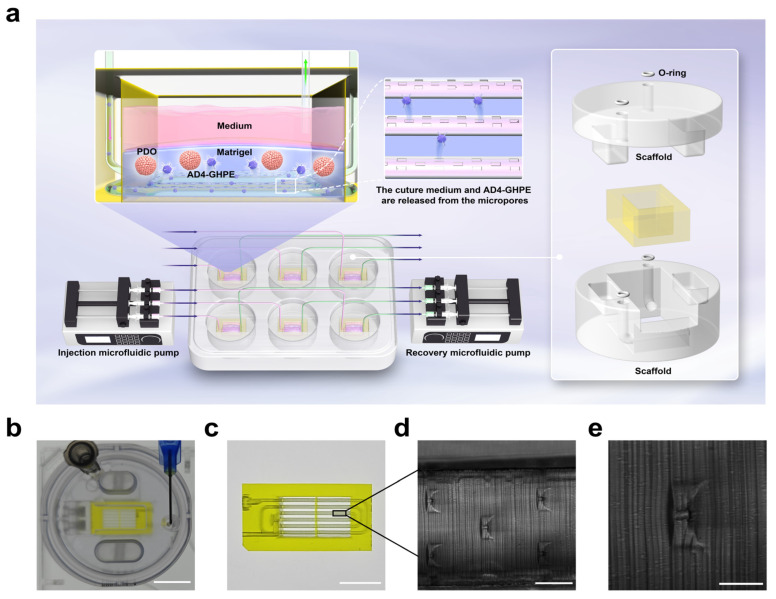
Microfluidic OOC systems structure. (**a**) Schematic diagram of the working principle of the microfluidic OOC system. Similar to the traditional method, PDOs are cultured in Matrigel. The microchannels at the bottom of the OOC deliver the culture medium and AD4-GHPE to the PDO through micropores. The OOC is fitted with an o-ring on the inlet and then clipped into the bottom half of the scaffold, which is designed to fit into a 6-well plate. The top half of the chip holder is placed on top to enclose the OOC. Tubing and needles are connected to the inlet and outlet ports on the lid and connected to their respective syringes. Two independently operable microfluidic pumps control the injection and aspiration of the syringes. These pumps control the flow of medium through the chip by simultaneously injecting medium through the inlet and aspirating waste through the outlet. (**b**–**e**) Physical detail pictures of the chip and the clamps. (**b**) The culture medium is injected into the microfluidic system of the chip through a blue L-shaped needle, and the supernatant is recovered through a black needle. (**c**) Top-down view of the chip. The chip has an open structure, and the PDOs are cultured in a square bioreactor. The microchannel structure responsible for transporting the culture medium is arranged in a spiral shape, and the culture medium flows out from the micropores and nourishes the PDO. (**d**,**e**) Micrographs of the internal structure of the chip. Scale bars: (**b**) 1 cm, (**c**) 70 μm, (**d**) 500 μm, (**e**) 50 μm.

In the subsequent development of the AD4-GHPE OV, experiments involving frequent liquid addition and supernatant extraction are prone to systematic errors due to human operation. We aimed to improve the automation and standardization of the experiments and reduce the operation time and steps by using a microfluidic OOC system. The schematic diagram of the system’s operation is shown in Figure 5a. We present a new 3D perfusion microchip model manufactured via microstereolithography 3D printing technology from BMF (Figure 5b–e). This structure is similar to previously reported structures [36], but has been modified with three rows of 7 µm open pores in the microchannels to ensure more uniform perfusion of PDOs embedded in matrix gel (Figure 5c). The OOC is fixed onto a six-well plate by two scaffolds. Each six-well plate can hold up to six OOCs, enabling batch operations. Since the chip has an open structure and the supernatant of the culture medium cannot be drawn out by pressure, we are equipped with two micro-pumps that can operate independently. Through a preset program, the culture medium enters the OOC through a syringe controlled by a micro-pump and perfuses the PDOs. Another micro-pump collects the culture medium supernatant.

### 3.6. AD4-GHPE Antitumor Experiment on the Chip

**Figure 6:**
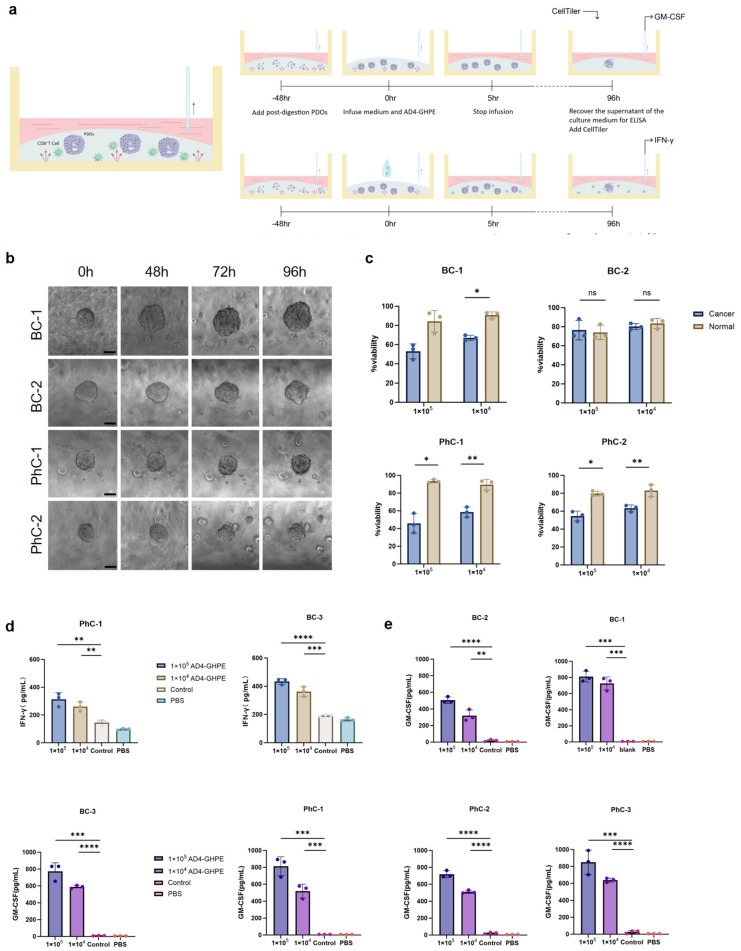
Exploring Three Tumor Killing Pathways of AD4-GHPE Oncolytic Virus Using the Microfluidics Chip System. (**a**) Experimental workflow diagram. PDOs were cultured on the chip for 48 h with slow infusion of medium through the microfluidic system before the experiment began. The PDOs were restored into complete 3D spheroids. From hours 0–5, media containing specific concentrations of AD4-GHPE (gradients of 1 × 10^5^ pfu/100 μL, 1 × 10^4^ pfu/100 μL, blank control) were infused, and the culture was continued for 96 h. At hour 96, the supernatants were collected for ELISA measurement and cell activity was assessed via CellTiter. (**b**) Representative images of BC-1, BC-2, PhC-1, and PhC-2 at hours 0, 48, 72, and 96. Scale bar: 100 μm. (**c**) After 96 h, cell viability was measured via CellTiter. The data are shown as the means ± SEMs, *n* = 3, ns ≥ 0.05, * *p* < 0.05, ** *p* < 0.05, *t* test. (**d**) After 96 h, the supernatants and cells were tested for IFN-γ expression via ELISA. The data are shown as the means ± SEMs, *n* = 3, ** *p* < 0.05, *** *p* < 0.001, **** *p* < 0.0001, *t* test. (**e**) After 96 h, the supernatants and cells were tested for GM-CSF expression via ELISA. The data are shown as the means ± SEMs, *n* = 3, ** *p* < 0.05, *** *p* < 0.001, **** *p* < 0.0001, *t* test. (**c**–**e**) Gradients were 1 × 10^5^ pfu/100 μL and 1 × 10^4^ pfu/100 μL.

Using this system, we continued the development of the AD4-GHPE OV. Following the same experimental approach and evaluation methods as those used in previous experiments, we cultured the granulated PDOs in the chip for 48 h to restore their full 3D spheroid structure (Figure 6a). For the specific cytotoxicity experiment, we used a microfluidic pump to inject a specific concentration of AD4-GHPE oncolytic virus into the organoid chip. After 96 h, we obtained cytotoxicity data for the four groups. Among the four PDO groups, three showed the same trends as in previous experiments (Figure 6b,c), with the AD4-GHPE OV showing significantly greater cytotoxicity in tumor PDOs than in normal tissue PDOs. However, in the BC-2 group, the survival rates of patients with tumor PDOs and normal tissue PDOs were not significantly different. We believe that this finding is due to individual patient heterogeneity, as the BC-2 tumor PDOs did not contain genes with matching TERTp promoters, preventing AD4-GHPE from replicating and leading to similar cytotoxicity.

To explore the feasibility of this system for coculturing CD8^+^ T cells and PDOs, we repeated the coculture experiments with BC-3 and PhC-1 cells. In the presence of the AD4-GHPE oncolytic virus, IFN-γ expression was significantly increased (Figure 6d). When the two sets of data were compared, the results revealed that, compared with traditional methods, microfluidic OOC technology yielded similar experimental outcomes. The mechanized operation of the OOC system reduces systematic errors and obtains a smaller variance than the traditional method, which has more definite statistical significance (Figure 3d, Figure 4a,b and Figure 6c,d). Moreover, compared with the traditional method, the OOC system saves the manual operations of adding culture medium and aspirating the supernatant through the preset program of the microfluidic pump, thus improving experimental efficiency.

In previous studies, GM-CSF has been shown to enhance antibody-dependent cellular cytotoxicity by stimulating neutrophils and plays an auxiliary role in the tumor-killing immune response. GM-CSF has been widely used in antitumor therapies [46,47]. We added a GM-CSF-overexpressing fragment to AD4-GHPE to achieve tumor killing via multiple pathways. In the in vitro cytotoxicity experiment, the microfluidic system automatically recovered the culture medium supernatant of the tumor PDOs. We performed GM-CSF ELISA and supplemented the data from BC-3 and PhC-3. The results revealed that the experimental group treated with the AD4-GHPE oncolytic virus presented significantly greater GM-CSF expression than did the control group (Figure 6e).

## 4. Discussion

Although significant progress has been made in new strategies for cancer treatment, such as immunotherapy, most patients with solid tumors still receive multimodal treatment involving surgery [48,49,50,51,52,53]. After surgery, patients often have various treatment options, including OV therapy. The PDO model established from surgical samples retains the biological characteristics of the patient’s primary tumor tissue, allowing us to provide personalized OV treatment recommendations. The microfluidics chip has helped us complete these experiments more efficiently and in a standardized manner. For example, in the PhC-1 experimental results, AD4-GHPE exhibited specific tumor cell-killing effects, and PD-L1 expression was detected on the surface of these tumor cells. AD4-GHPE reduced PD-L1 expression and enhanced the T-cell antitumor response. Additionally, GM-CSF was significantly expressed. On the basis of the experimental data, we suggest that AD4-GHPE is a viable treatment option for patients. In contrast, in the BC-2 experimental results, AD4-GHPE did not show specificity in its cytotoxicity in tumor organoids or normal tissue organoids. We attribute this to the absence of genes in BC-2 tumor PDOs that match the TERTp promoter of AD4-GHPE. This absence prevents AD4-GHPE from undergoing specific viral replication. Consequently, AD4-GHPE fails to generate specific cytotoxicity, and the antitumor pathways of reducing PD-L1 expression and secreting GM-CSF cannot be effectively realized. For patients with similarities to BC-2, we suggest that other therapeutic options should be considered instead of AD4-GHPE treatment. It can be stated that the interindividual differences among patients have a decisive impact on the therapeutic effect of AD4-GHPE, which also reflects the necessity of personalized testing.

As emerging cancer therapies, OVs offer advantages including good targeting abilities, minimal side effects, and a low likelihood of resistance development. Four OVs have been approved for marketing, and related clinical trial data are continuously being obtained, increasing the popularity of OV development. However, they still have limitations that cannot be ignored. To ensure the safety of oncolytic viruses, the vast majority of researchers tend to select viruses with relatively weak pathogenicity for improvement, such as Adenovirus (AdV), Herpes simplex virus (HSV), and Vaccine virus (VACV). This also results in the relatively weak tumor-killing ability of oncolytic viruses observed when they are used alone. This issue also occurs with AD4-GHPE. At a concentration of 1 × 10^5^, the survival rate of tumor PDOs still ranges from 45.61% to 56.30%. Currently, the main solution to this problem is to increase the tumor-killing pathways of oncolytic viruses. Among the novel OVs being developed, the encoding of specific cytokines to stimulate tumor immune responses is an important research direction, with IL-7, IL-12, IL-15 [54,55], and others representing key candidates. In this study, we encoded GM-CSF in the AD4-GHPE oncolytic virus and demonstrated its high expression in tumor organoids. During the experiment, we used a microfluidic OOC platform to automate the perfusion of culture media and AD4-GHPE while collecting the cell supernatant as samples, thus reducing operational steps and human errors. Moreover, this system has the advantages of low learning costs and adaptability to various primary tumor types. To verify the approach, we used PDOs derived from breast cancer and hypopharyngeal cancer patients, both of which yielded reasonable experimental data. The microfluidic OOC provides a mechanized and automated validation platform for OVs.

The microvasculature is an essential feature of almost all human tissues; tumor tissues also contain microvasculature. The recapitulation of tumor tissue supply patterns, or vascularization in tumor organoids, has become a research focus [56]. By setting up microchannels that simulate the tumor microvasculature, the system can handle the exchange of nutrients, metabolites, and large quantities of biochemical molecules during various physiological processes [57,58,59]. The culture chamber of our microfluidic organoid chip model features a spiral tubular structure, with three microholes spaced every 70 µm along the tubular structure, enabling efficient and convenient perfusion of fluid into the PDOs. The microhole diameter is 7 µm, similar to that of capillaries in the human body. Although we have simulated the capillary diameter and physical structure, we have not yet laid out endothelial precursors or endothelial cells; thus, further work is needed to produce a true capillary-like organ chip. We plan to improve the vascularization aspect in subsequent experiments. Currently, the advantages of this microfluidic organoid system lie in its convenience and low learning cost. When dealing with PDOs from multiple tumor specimen sources, we can directly apply media formulations and coculture methods from traditional culture protocols for experimentation.

In conclusion, we report a novel oncolytic virus, AD4-GHPE, which we validated on a microfluidic organoid chip platform, demonstrating its excellent capabilities in three antitumor strategies and highlighting the enormous potential of the microfluidic OOC in developing oncolytic viruses with complex functions. We hope that our study provides an automated and standardized approach for oncolytic virus development.

## Data Availability

The datasets used and/or analyzed during the current study are available from the corresponding author upon reasonable request.
